# Prevalence and Outcomes of HER2-Low Versus HER2-0 Status in Patients with Metastatic Breast Cancer

**DOI:** 10.3390/cancers18020253

**Published:** 2026-01-14

**Authors:** Akshara Singareeka Raghavendra, Diane D. Liu, Senthil Damodaran, Sarah Pasyar, Yu Shen, Jason A. Mouabbi, Carlos H. Barcenas, Kelly K. Hunt, Debu Tripathy

**Affiliations:** 1 Department of Breast Medical Oncology, Division of Cancer Medicine, The University of Texas MD Anderson Cancer Center, Houston, TX 77030, USA; 2 Department of Biostatistics, The University of Texas MD Anderson Cancer Center, Houston, TX 77030, USA; 3 Department of Breast Surgical Oncology, The University of Texas MD Anderson Cancer Center, 1515 Holcombe Blvd., Houston, TX 77030, USA

**Keywords:** HER2-low, metastatic, de novo, prevalence, overall survival, breast cancer

## Abstract

**Background:** HER2-low breast cancer (HER2 immunohistochemical [IHC] score 1+, or IHC 2+ without HER2 gene amplification) is distinct from HER2-positive and HER2-0 breast cancer (IHC 0), with a differing prognosis and specific therapeutic options. The DESTINY-Breast04 trial demonstrated notable efficacy of the HER2 antibody–drug conjugate trastuzumab deruxtecan over standard chemotherapy in patients with metastatic breast cancer (MBC) defined as HER2-low. More recently, the DESTINY-Breast06 trial confirmed this benefit in hormone receptor-positive and HER2-ultralow (less than 1+, but with ≤10% of infiltrating cancer cells showing incomplete and faint/weak membrane staining) cases, prompting re-evaluation of HER2 diagnostic thresholds and treatment strategies. **Methods**: Eligible patients were women with HER2-low or HER2-0 MBC evaluated at MD Anderson between January 2006 and January 2019. HER2-low was defined as either (1) IHC 1+ or (2) IHC 2+ and negative on fluorescence in situ hybridization. Multivariate logistic regression was used to evaluate distinct clinicopathologic features of patients with HER2-low status. Overall survival (OS) was estimated by the Kaplan–Meier method. Multivariate Cox proportional hazards regression was applied to assess the effects of covariates of interest on OS across different HER2 groups. **Results**: We included 3834 women: 2637 (69%) with recurrent and 1197 (31%) with de novo MBC; HER2-low disease was present in 1575 (60%) and 712 (59%), respectively. In de novo cases, higher nuclear grade was associated with HER2-low status (grade 2 vs. 1, OR = 2.02, *p* = 0.007; grade 3 vs. 1, OR = 1.87, *p* = 0.015), while recurrent cases were associated with ER-positivity (OR = 1.96, *p* < 0.001) and prior adjuvant radiotherapy (OR = 0.79, *p* = 0.007). Median OS was 3.2 years (95% CI 3.0–3.5). In de novo disease, Black race (HR = 1.48), metaplastic (HR = 3.15) or other non-ductal/lobular histologies (HR = 2.36), and grade 3 (HR = 1.67) predicted worse OS, whereas Hispanic ethnicity (HR = 0.74) and Other races (HR = 0.57), higher ER (HR = 0.48–0.41) and PR (HR = 0.72–0.53), and HER2-low status (HR = 0.77) conferred improved outcomes. In recurrent disease, Black race predicted worse OS (HR = 1.21, 95% CI 1.05–1.39), while Other race (HR = 0.78, 95% CI 0.62–0.97), higher ER (HR = 0.69–0.44) and PR (HR = 0.73–0.73), and HER2-low (HR = 0.89) were protective. HER2 discordance between primary and metastatic sites occurred in 38.8% of recurrent and 13.1% of de novo cases. **Conclusions**: HER2-low status was significantly associated with longer OS compared to HER2-0 status in both recurrent and de novo MBC cases. These real-world data help establish the prevalence of HER2-low status and its distinct outcomes. The discrepancy in HER2-low status between the primary tumor and metastatic sites highlights the potential for changes in HER2 expression over time, exploring the interaction between HER2-low breast cancer and the tumor microenvironment and emphasizing the importance of monitoring and reassessing HER2 status at various stages to guide treatment decisions effectively and the need for more quantitative and reproducible HER assays.

## Simple Summary

Breast cancers with low levels of HER2 expression (HER2-low) represent a large proportion of tumors traditionally classified as HER2-negative and have recently become clinically relevant with the availability of effective HER2-targeted antibody–drug conjugates. In this large, real-world cohort of patients with metastatic breast cancer, we found that HER2-low disease was common in both de novo and recurrent metastatic settings and was associated with longer overall survival compared with HER2-0 disease. We also observed frequent changes in HER2 status between primary tumors and metastatic sites, particularly in recurrent disease, highlighting tumor heterogeneity and the dynamic nature of HER2 expression over time. These findings support routine reassessment of HER2 status in metastatic breast cancer prior to changing therapy when feasible and underscore the need for more accurate and reproducible methods to measure low levels of HER2 expression.

## 1. Introduction

Breast cancer is a complex and heterogeneous disease with various cell receptor and molecular subtypes that have distinct characteristics and responses to treatment. Human epidermal growth factor receptor 2 (HER2)-low breast cancer, defined by low levels of HER2 protein expression (measured by immunohistochemical [IHC] analysis) in classically HER2-negative tumors, ref. [1] has emerged as an important subtype, challenging the traditional understanding of HER2 status as binary (positive or negative).

Recent multi-omics and real-world studies [2,3] suggest HER2-low tumors comprise ~55–65% of all HER2-negative breast cancers, though classification remains variable due to poor reproducibility between pathologists (with agreement between IHC 0 and 1+ as low as 25–30%).

Current HER2 diagnostic tests were designed to identify cancers most sensitive to treatment with trastuzumab, a monoclonal antibody that targets HER2, and thus are optimized for cancers with high levels of HER2 overexpression [4]. However, evidence from a phase 3 study (DESTINY-Breast04) demonstrated the efficacy of the antibody–drug conjugate trastuzumab deruxtecan (T-DXd) in a proportion of patients with tumors classified as HER2-negative, suggesting the need for diagnostics that can identify patients who may benefit from novel HER2-targeted therapies that demonstrate efficacy at levels of HER2 expression lower than those currently defined as HER2 positivity [5]. Findings from the DESTINY-Breast06 trial [6] further validated HER2-low as a therapeutically relevant classification, especially in hormone receptor-positive (HR+) metastatic breast cancer (MBC), [7,8] emphasizing the need for consistent, accurate HER2 IHC interpretation and the development of novel companion diagnostics. Since the US Food and Drug Administration (FDA) granted approval for T-DXd in patients with HR+/HER2-ultralow metastatic breast cancer on 27 January 2025, the distinction between HER2-0 and HER2-ultralow has become clinically relevant.

The *HER2* gene, also known as *ERBB2*, encodes the HER2 receptor protein, a transmembrane tyrosine kinase receptor that plays a crucial role in regulating cell growth, differentiation, and survival. Normal breast epithelial cells typically have one copy of the *HER2* gene on each chromosome 17 and express HER2 protein at detectable levels [9]. When amplified or overexpressed, the *HER2* gene drives oncogenic signaling that promotes proliferation, survival, and malignant transformation in breast cancer [10]. Historically, HER2-positive breast cancer, representing about 20% of all breast cancer cases, is characterized by high HER2 protein expression [11] and has been associated with aggressive tumor behavior and poorer clinical outcomes.

Breast cancer with low levels of HER2 protein expression below the positivity threshold represents a substantial proportion of cases, estimated to be around 40–50%, [12] and was previously categorized as HER2-negative. However, HER2-low breast cancer exhibits unique molecular characteristics that distinguish it from both HER2-positive and HER2-negative subtypes. Gene expression profiling studies [13,14] have revealed differences in the genomic aberrations like copy number alterations and transcriptomic profiles of HER2-low breast tumors, indicating distinct signaling pathways; however, some studies [15,16] have shown no genomic difference between HER2-0 and HER2-low tumors. This heterogeneity suggests that distinct mechanisms may drive the growth and progression of HER2-low breast cancer, requiring tailored treatment strategies.

The main goals of this study were to measure the prevalence of HER2-low status (IHC 1+ or IHC 2+ and fluorescence in situ hybridization [FISH] negative) in a large cohort of patients with MBC, identify clinicopathological/treatment associations, and compare overall survival (OS) between patients with HER2-0 (IHC 0) and HER2-low MBC. Furthermore, this study characterized the discrepancy in HER2 status between the primary tumor and metastatic lesions, as well as temporal variations in HER2 expression across multiple time points.

## 2. Patients and Methods

### 2.1. Patient Population

The Breast Cancer Management System database of the Department of Breast Medical Oncology encompasses a comprehensive record of breast cancer patients referred to The University of Texas MD Anderson Cancer Center since the 1990s. This database was queried to include patients aged 18 or older at diagnosis and evaluated at MD Anderson between January 2006 and January 2019 with a confirmed diagnosis of recurrent or de novo stage IV MBC with HER2-0 or HER2-low expression in the primary breast tumor, regardless of HR status. Patients with recurrent MBC were initially treated for stage I-III breast cancer with later (more than 90 days after diagnosis) development of distant recurrence, while de novo MBC was defined as stage IV breast cancer identified within 90 days of initial diagnosis; these groups were differentiated for the purposes of a sensitivity analysis. The HER2 status was determined using either IHC or gene amplification using fluorescence in situ hybridization (FISH) to assess the presence of HER2 gene amplification and the corresponding protein expression. HER2-0 expression was defined as IHC 0, while HER2-low was defined as either (1) IHC 1+ or (2) IHC 2+ and negative for gene amplification on FISH. HER2-ultralow cases were not called out, as this analysis antedated the results of the DESTINY-Breast06 trial.

The collected demographic, clinical, and pathological characteristics encompassed age, race/ethnicity, disease stage, clinical nodal status, sites of metastasis, history of neoadjuvant and/or adjuvant treatments, prior lines of systemic treatments in the metastatic setting, and disease status at the time of last contact or death. The clinical stages, evaluated at the initial diagnosis or at the time of distant recurrence, were determined in accordance with the American Joint Committee on Cancer guidelines that were current at the time of diagnosis. Biopsy of metastatic sites was performed based on clinical indication criteria, which included site accessibility, diagnostic confirmation, and suspected clinical progression. In addition to HER2 status, HR positivity was defined based on the expression of estrogen receptor (ER) or progesterone receptor (PR), as determined by standard IHC using institutional cutoffs that adhered to the guidelines set by the American Society of Clinical Oncology and the College of American Pathologists (ASCO/CAP) [11].

### 2.2. Statistical Analysis

Data were summarized using descriptive statistics such as mean, standard deviation, median, and range for continuous variables and frequency and proportion for categorical variables. The association between categorical variables was examined by the chi-squared test or Fisher exact test when appropriate. The Wilcoxon rank sum test was used to examine the differences in continuous variables between two groups. A logistic regression model was used to estimate the association between demographic and clinicopathological characteristics and HER2-negative status. OS time was defined as the duration from the date of diagnosis of metastatic disease until death from any cause. A patient was censored if alive at the time of the last follow-up. OS times were estimated using the Kaplan–Meier method and compared between patient-characteristic groups by the log-rank test. A multivariate Cox proportional hazards regression model was applied to assess the effect of covariates of interest on OS. McNemar’s test was used to assess whether the percentages of patients with HER2-low status differed significantly between the primary tumor and the metastasis site, without adjustments for multiple comparisons. All computations were carried out in SAS 9.4.

## 3. Results

### 3.1. Patient Characteristics

This study included 3834 patients with HER2-low or HER2-0 MBC, of which 2637 (69%) patients had recurrent disease and 1197 (31%) had de novo MBC. Among these groups, 1575 (60%) and 712 (59%) patients exhibited HER2-low status, respectively. Table 1 presents the demographic and clinicopathological characteristics of the recurrent disease group stratified into HER2-0 (IHC 0) and HER2-low (IHC 1+ or IHC 2+/FISH−) groups. In multivariate logistic regression analysis, HER2-low status was significantly associated with initial clinical stage (III vs. I, odds ratio [OR] = 1.42, *p* = 0.005), ER-positivity in the primary tumor (OR = 1.96, *p* < 0.001), and use of adjuvant radiation therapy (OR = 0.79, *p* = 0.007) (Table 2).

Table 3 shows the demographic and clinicopathological characteristics of the de novo MBC group stratified into HER2-0 and HER2-low groups. In univariate analysis, HER2-low status was associated with post-menopausal status, higher nuclear grade (2 and 3 vs. 1), and HR+ status (indicated by 55–100% of tumor nuclei being ER-positive). In multivariate logistic regression analysis, the only variable significantly associated with HER2-low status was higher nuclear grade (grade 2 [vs. 1], OR = 2.016, *p* = 0.0068; grade 3 [vs. 1], OR = 1.869, *p* = 0.0145) (Table 4).

### 3.2. Overall Survival

**Figure 1:**
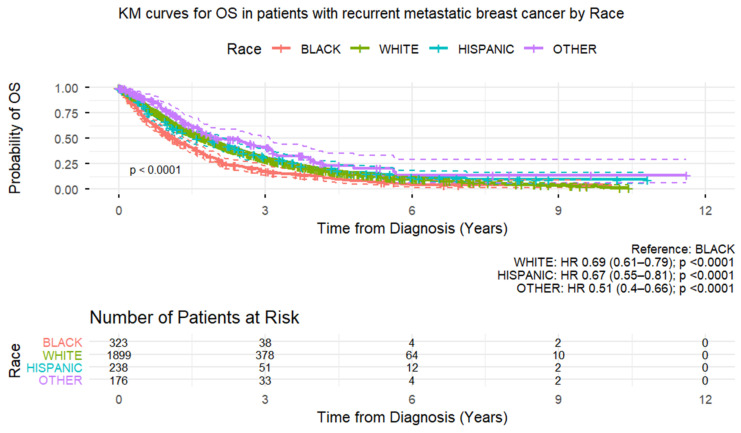

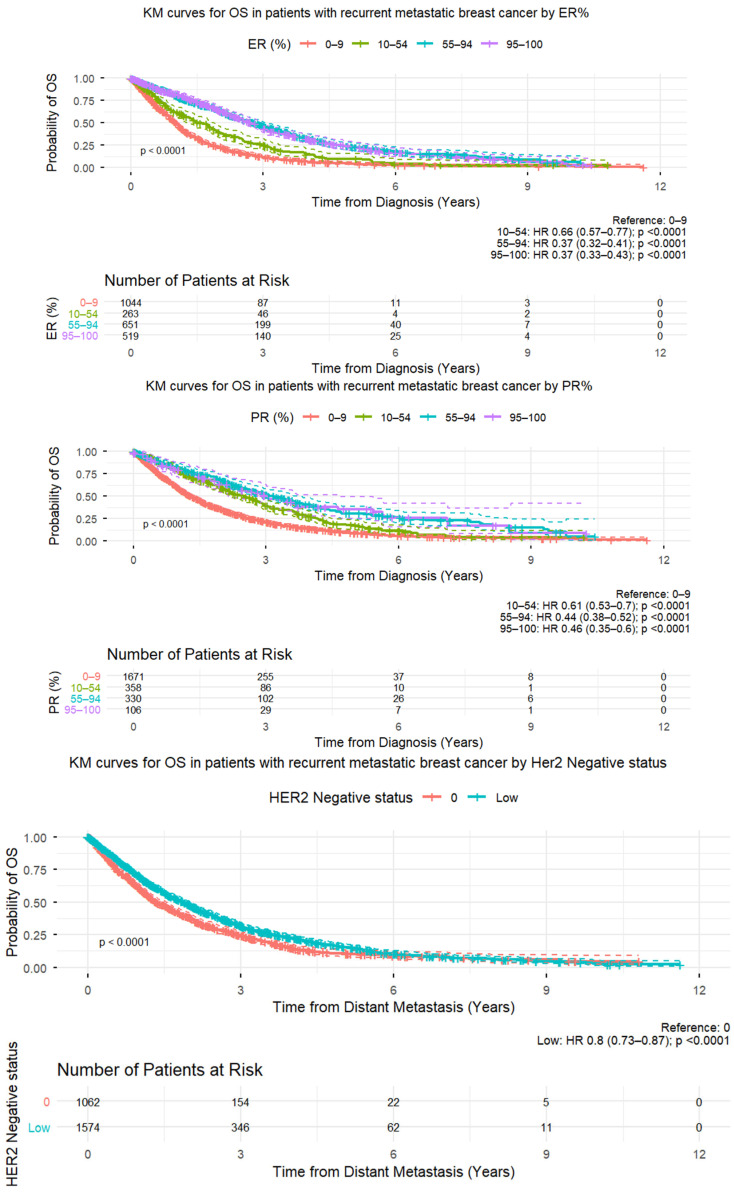
Kaplan–Meier curves of overall survival in patients with recurrent metastatic breast cancer.

With a median follow-up of 5.7 years, 767 (64%) patients died among the de novo MBC patients. For the recurrent MBC group, with a median follow-up of 4.3 years, 1892 (72%) patients died. The median OS was 3.2 years (95% CI: 3.0–3.5 years) in the de novo MBC group and 1.6 years (95% CI: 1.5–1.8 year) in the recurrent MBC group. In the recurrent MBC group, Supplement Table S1 shows landmark survival rates at 2 and 5 years post-diagnosis within each of the demographic and clinicopathological categories. In the recurrent MBC group, significant predictors of shorter OS time were seen only in the 2-year timeframe and included Black race, higher nuclear grade, presence of lymphovascular invasion, hormone receptor-low/negative, and HER2-negative status. In the multivariate Cox proportional hazards regression model for OS in the recurrent MBC group, shorter OS time was significantly associated with Black race (hazard ratio [HR] = 1.210 [95% CI 1.051–1.392], *p* = 0.0078), lower ER and PR expression (staining percentage) (ER ≥ 95% [vs. 0–9%] HR = 0.439 [95% CI 0.376–0.513], *p* < 0.0001; PR ≥ 95% [vs. 0–9%] HR = 0.728 [95% CI 0.547–0.969], *p* = 0.029), and HER2-negative status (HR = 0.890 [95% CI 0.807–0.981], *p* = 0.0194) (Table 5 and Figure 1).

**Figure 2:**
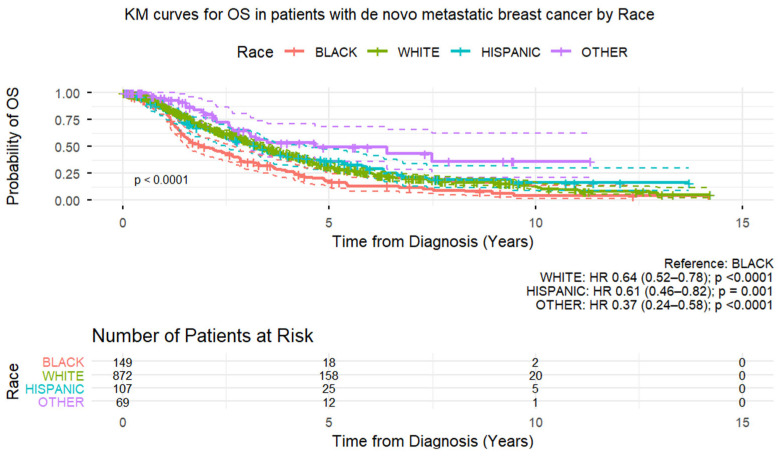

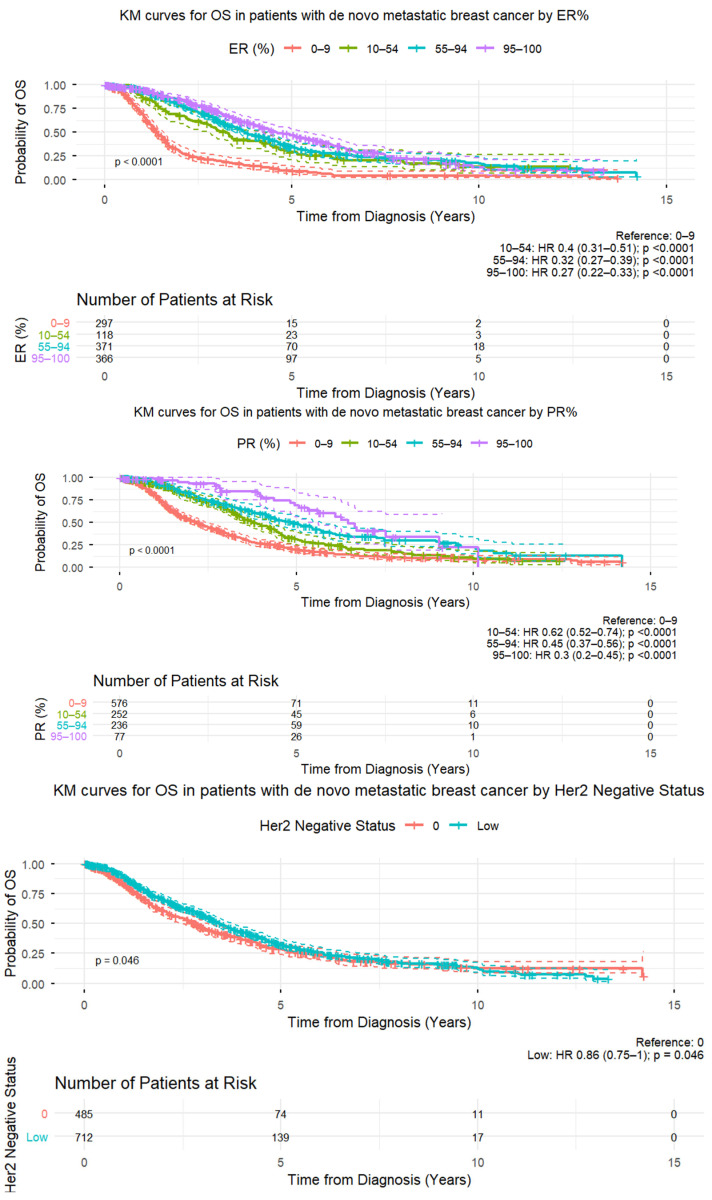

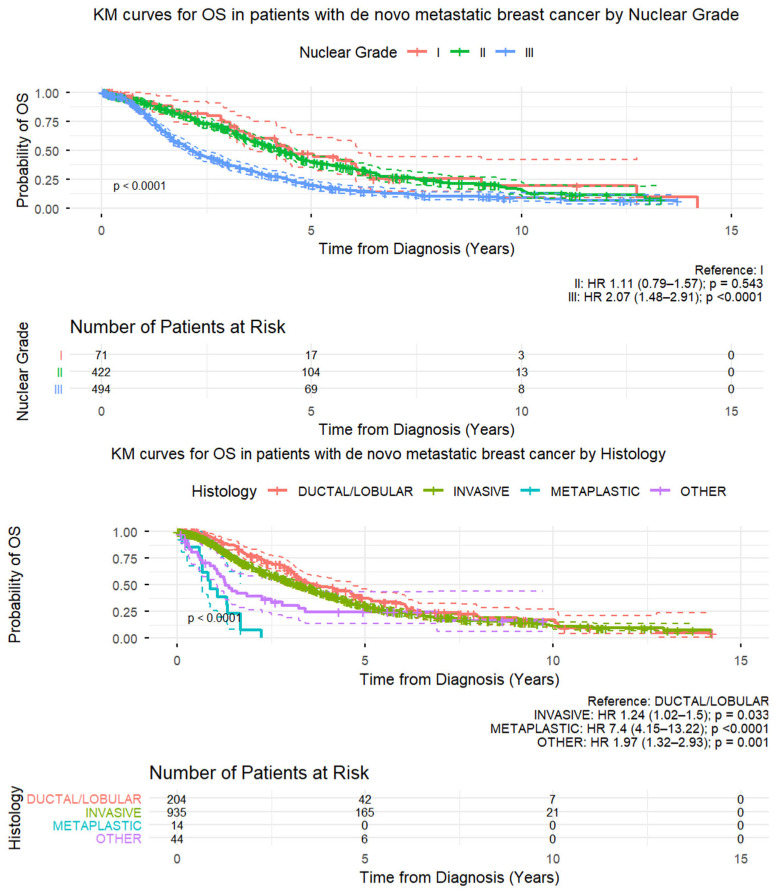
Kaplan–Meier curves of overall survival in patients with de novo metastatic breast cancer.

In the de novo MBC group, from Supplement Table S2, significant predictors of shorter OS time were seen only in the 1-year timeframe and included Black race, metaplastic histology, higher nuclear grade, presence of lymphovascular invasion, and hormone receptor-negative status. In the multivariate Cox proportional hazards regression model for OS in the de novo MBC group, shorter OS time was significantly associated with Black race (HR = 1.483 [95% CI 1.183–1.859], *p* = 0.0006), metaplastic histology (HR = 3.146 [95% CI 1.592–6.218], *p* = 0.0010), higher nuclear grade (grade 3 [vs. 1] HR = 1.673 [95% CI 1.145–2.446], *p* = 0.0079), lower ER and PR expression (staining percentage) [ER ≥ 95% [vs. 0–9%] HR = 0.413 [95% CI 0.318–0.536], *p* < 0.0001; PR ≥ 95% [vs. 0–9%] HR = 0.527 [95% CI 0.338–0.822], *p* = 0.0048), and HER2-negative status (HR = 0.774 [95% CI 0.656–0.913], *p* = 0.0025) (Table 6 and Figure 2).

### 3.3. Discordance in HER2 Status Between Primary and Metastatic Tumors

HER2-low status was, in general, more common in primary tumors than in metastases. Among the patients with recurrent MBC after a prior diagnosis of early-stage breast cancer, the proportion with HER2-low status (vs. HER2-0) decreased from 61.4% in the primary tumor to 48.3% in the metastasis site (McNemar’s test *p* < 0.0001) (Table 7). Similarly, among those with de novo MBC, 54.9% had HER2-low disease at the primary site and 50.0% had HER2-low disease at the site of metastasis (McNemar’s test *p* = 0.0078) (Table 7). Among the 609 recurrent cases, 236 (38.75%) underwent a change—that is, showed discordance—in HER2 status upon distant recurrence, with 158 gaining and 78 losing HER2 expression. This difference was less pronounced in the de novo MBC group, as among 388 in the de novo group, 51 had a change in the HER2 status in the metastasis (a discordance of 13.14%) (Table 7).

### 3.4. Types of Metastases and the Discordance in HER2 Status with Primary Tumors

We examined HER2 status by types and locations of metastases among those whose HER2 status changed. Non-visceral metastasis was more frequent than visceral metastasis (62.4% non-visceral vs. 37.6% visceral in recurrent MBC and 70.1% vs. 29.9%, respectively, in de novo MBC), and bone was the most frequent metastasis site (40.6% in recurrent and 55.4% in de novo MBC) (Table 8).

Among the MBC recurrent cases, the prevalence of HER2-low status was not different between visceral and non-visceral metastasis. Among the 229 patients (37.6% of the recurrent MBC group) with visceral metastasis, 143 (62.5%) had HER2-low primary tumors and 108 (47.2%) had HER2-low metastatic tumors (*p* < 0.0001). Among the 380 (62.39%) with non-visceral metastasis, 231 (60.8%) had a HER2-low primary tumor, and 186 (48.9%) had a HER2-low metastatic tumor (*p* = 0.0003) (Table 9).

In the de novo MBC group, the 116 (29.9%) patients with visceral metastasis showed notable similarity between primary and metastatic tumors, as 56.0% had HER2-low primary tumors and 51.7% had HER2-low metastatic tumors (*p* = 0.1655). Among the 272 (70.1%) with non-visceral metastasis, 60.8% had a HER2-low primary tumor and 49.3% had a HER2-low metastatic tumor (*p* = 0.0231) (Table 10).

## 4. Discussion

In patients with recurrent MBC and patients with de novo MBC, HER2-low status was significantly associated with a longer OS compared to HER2-0 status. Initial disease stage, ER-positivity, and use of adjuvant radiation therapy were associated with HER2-low status in the recurrent MBC group, while higher nuclear grade was significantly associated with HER2-low status in the de novo MBC group. Also, a notable discrepancy in HER2 status was observed between the primary breast cancers and distant metastases. This correlation suggests a potential link between the biological characteristics of the primary tumor and the development of recurrent metastatic disease in the context of HER2 status in individuals with early-stage or de novo MBC. These differences in phenotypes and outcomes between HER2-0 and HER2-low status could be, in part, due to the association between HER2-0 with ER-positivity; however, these differences persisted in the multivariate analysis that included ER.

The phase 3 DESTINY-Breast06 trial [6] demonstrated superior progression-free survival with trastuzumab deruxtecan versus standard chemotherapy in patients with HR+ HER2-low (and -ultralow) MBC, redefining therapeutic thresholds. Importantly, this trial included a central pathology review, helping to mitigate the variability in HER2 classification that has limited prior real-world comparisons.

Emerging data [17] suggest further stratification within the HER2-low group, with ultralow (IHC >0 but <1+) tumors showing intermediate response rates to HER2-directed antibody–drug conjugates. This may prompt future updates to IHC scoring criteria and therapeutic algorithms.

Recent studies [18,19] have confirmed substantial intrapatient heterogeneity and HER2 status evolution, particularly in response to systemic therapy.

HER2-low breast cancer has unique molecular characteristics and clinical behavior that distinguish it from both HER2-positive and HER2-negative disease [12]. The precise definition of HER2-low breast cancer is still evolving, and there is currently no universally accepted formal definition [9]. Conventionally, HER2-low breast cancers are defined based on an IHC score of 1+ or an IHC score of 2+ with a negative result on in situ hybridization [9].

The concept of HER2-low emerged as a response to the realization that some breast cancers have HER2 expression levels that are not high enough to be considered HER2-positive but may still have the potential to respond to certain therapies, particularly those targeting HER2. Prior analyses have shown that within the traditional HER2-negative category, there is heterogeneity in HER2 expression [20]. The HER2-low category has emerged as a result of improvements in the detection and quantification of HER2 expression as well as the development of novel antibody–drug conjugates that are posited to have both direct and bystander effects through targeting the surrounding tumor microenvironment, thus affecting cells with lower or no HER2 expression on IHC, thereby increasing the therapeutic impact [1].

The legacy HER2 IHC assay was developed as a companion diagnostic for ERBB2-targeting monoclonal antibodies such as trastuzumab. However, there exists significant discordance and less reliability between pathologists in scoring IHC 0, 1+, and 2+ cases [21]. Furthermore, this study’s results align with findings from other studies that employed different methodologies, indicating a high level of disagreement among pathologists when scoring HER2 IHC 0 versus non-0 cases. Specifically, the ONEST (Observers Needed to Evaluate Subjective Tests) method reported a substantial 40.6% disagreement in this regard [22]. These findings suggest that the legacy HER2 IHC assay is likely to be inaccurate and insufficient for making clinical decisions, particularly in prescribing HER2-low-specific treatments like trastuzumab deruxtecan [23]. Research is ongoing to better understand and quantify HER2-low breast cancer and develop effective treatment strategies for this subtype, especially advanced/metastatic cases [9]; thus, the lack of standardization in the classification of HER2-low breast cancers also poses challenges to obtaining accurate and reproducible results [9]. Improved histological assessment using immuno-fluorescent HER2 staining or other quantitative techniques is needed and is in the process of being prospectively tested in the TBCRC 066 trial [24]. In another study, involving 18 pathologists, breast cancer biopsies examined using the standard IHC scale were rated with only a 26% agreement between scores 0 and 1+; a higher concordance of 58% was found between scores 2+ and 3+ [21].

The classification of HER2-low breast cancer has significant clinical implications, as it may require alternative treatment approaches compared to HER2-positive breast cancer. While HER2-positive breast cancers have been associated with higher stage, positive lymph nodes, higher proliferation rate, and lack of expression of hormone receptors, traditional HER2-targeted therapies, such as trastuzumab have shown limited efficacy in HER2-low breast cancer [25]. However, more potent or higher affinity HER2-targeted therapies are showing activity in HER2-low breast cancer. These agents include new antibodies, small-molecule inhibitors, and immune-based therapies that may exploit the unique molecular characteristics of HER2-low tumors. In a phase 3 trial (DESTINY-Breast04) [26] involving patients with HER2-low MBC, treatment with trastuzumab deruxtecan showed a median progression-free survival of 10.1 months compared to 5.4 months with the physician’s choice of treatment (HR = 0.51, *p* < 0.001). Trastuzumab deruxtecan also showed significantly longer OS at 23.9 months (about 2 years) vs. 17.5 months (HR = 0.64, *p* = 0.003). The phase II DAISY trial [27] demonstrated that trastuzumab deruxtecan (T-DXd) achieved high activity across varying levels of HER2 expression, with objective response rates of 70.6% in patients with HER2-overexpressing disease, 37.5% in HER2-low, and 29.7% in HER2-0 metastatic breast cancer (N = 177). While responses correlated with HER2 expression and drug uptake, activity in some HER2-0 tumors suggested that additional, HER2-independent mechanisms may contribute to efficacy. Additionally, understanding the interplay between HER2-low breast cancer and the tumor microenvironment may provide insights into the potential role of immunotherapy in this subtype [28].

Our study found a 38.75% discordance in the recurrent MBC group and a 13.14% discordance in the de novo MBC group in HER2-low status (vs. HER2-0) between primary and metastatic sites. The discordance between primary and metastatic HER2 status can be due to several factors, including interobserver variability among pathologists, [21] tumor heterogeneity and subclonal selection over time under selective pressure, and a drift to ER-negative and/or HER2-negative status because of exposure to therapy [29,30,31]. This discordance highlights the need for careful evaluation of recurrent/progressive disease, particularly more rapidly proliferating lesions. Interestingly, there were lesser differences between primary and metastatic HER2-low status found in the de novo MBC group, both in the visceral metastasis subset and in the entire de novo MBC group.

Limitations of our study include its retrospective nature and lack of central pathology review, although all cases were reviewed by pathologists at our institution. Central pathology review is particularly relevant for HER2-low classification, as prior studies have demonstrated interobserver variability in distinguishing IHC 0 from 1+, which may have influenced prevalence estimates and observed HER2 discordance. Interpretation of HER2 discordance should also account for clinical and treatment-related factors. Intervening systemic therapies may influence HER2 expression over time; however, limited granularity regarding treatment timing and sequencing precluded formal modeling of predictors of HER2 status change. Accordingly, discordance analyses stratified by disease presentation (recurrent versus de novo) and hormone receptor status should be interpreted as exploratory. Although multivariable models were adjusted for key clinicopathologic variables, treatment-related confounding during the metastatic phase could not be fully addressed. As a result, the observed association between HER2-low status and improved overall survival may reflect, in part, underlying hormone receptor biology and treatment responsiveness rather than a purely independent prognostic effect. Finally, subgroup analyses were not adjusted for multiple comparisons and should be considered hypothesis-generating. Missing covariates, including menopausal status, body mass index, and lymphovascular invasion were handled using available-case analyses, and residual bias due to missing data is possible. In addition to the absence of central pathology review, this study did not incorporate AI-based HER2 scoring tools, which have shown promise in reducing interobserver variability and improving identification of HER2-low cases.

Going forward, these findings underscore the importance of accurate HER2 classification, reassessment of HER2 status at diagnosis of metastatic recurrence, and further prospective validation of treatment approaches—especially those involving antibody–drug conjugates—in HER2-low metastatic breast cancer.

## 5. Conclusions

In this large real-world cohort of metastatic breast cancer, HER2-low disease was common and was associated with longer overall survival compared with HER2-0 disease in both de novo and recurrent settings. Clinically meaningful discordance in HER2 status between primary and metastatic tumors was observed, underscoring the dynamic nature of HER2 expression. These findings support reassessment of HER2 status at metastatic progression and reinforce the clinical relevance of HER2-low breast cancer in the era of HER2-targeted antibody–drug conjugates.

## Figures and Tables

**Table 1:** Demographic and clinicopathological characteristics by HER2 status in patients with recurrent metastatic breast cancer.

		All Patients with Recurrence (*n* = 2637), *n* (%)	HER2 Status		
Covariate			HER2-0 (*n* = 1062), *n* (%)	HER2-Low (*n* = 1575), *n* (%)	Univariate *p*-Value
Age	≤40	575 (21.8%)	226 (21.3%)	349 (22.2%)	0.242
	41–59	1456 (55.2%)	608 (57.3%)	848 (53.8%)	
	60–69	423 (16.0%)	154 (14.5%)	269 (17.1%)	
	≥70	183 (6.9%)	74 (7.0%)	109 (6.9%)	
Race and ethnicity	Black	323 (12.2%)	142 (13.4%)	181 (11.5%)	0.021
	Hispanic	238 (9.0%)	101 (9.5%)	137 (8.7%)	
	Other	176 (6.7%)	86 (8.1%)	90 (5.7%)	
	White	1900 (72.1%)	733 (69.0%)	1167 (74.1%)	
Body mass index	Normal	796 (30.2%)	330 (31.1%)	466 (29.6%)	0.334
	Obese	817 (31.0%)	308 (29.0%)	509 (32.3%)	
	Overweight	766 (29.0%)	319 (30.0%)	447 (28.4%)	
	Underweight	40 (1.5%)	17 (1.6%)	23 (1.5%)	
	Unknown	218 (8.3%)	88 (8.3%)	130 (8.3%)	
Menopausal status	Post	972 (36.9%)	353 (33.2%)	619 (39.3%)	0.865
	Pre	785 (29.8%)	293 (27.6%)	492 (31.2%)	
	Other	32 (1.2%)	11 (1.0%)	21 (1.3%)	
	Unknown	848 (32.2%)	405 (38.1%)	443 (28.1%)	
Clinical stage	I	405 (15.4%)	178 (16.8%)	227 (14.4%)	0.088
	II	1239 (47.0%)	507 (47.7%)	732 (46.5%)	
	III	993 (37.7%)	377 (35.5%)	616 (39.1%)	
Histology	Invasive ductal	2236 (84.8%)	897 (84.5%)	1339 (85.0%)	0.001
	Invasive lobular	223 (8.5%)	81 (7.6%)	142 (9.0%)	
	Metaplastic	52 (2.0%)	34 (3.2%)	18 (1.1%)	
	Invasive mixed ductal/lobular	77 (2.9%)	26 (2.4%)	51 (3.2%)	
	Other	49 (1.9%)	24 (2.3%)	25 (1.6%)	
Nuclear grade	I	72 (2.7%)	34 (3.2%)	38 (2.4%)	0.012
	II	744 (28.2%)	259 (24.4%)	485 (30.8%)	
	III	1493 (56.6%)	601 (56.6%)	892 (56.6%)	
	Unknown	328 (12.4%)	168 (15.8%)	160 (10.2%)	
Lymphovascular invasion of primary tumor	Positive	927 (35.2%)	346 (32.6%)	581 (36.9%)	0.673
	Negative	1193 (45.2%)	456 (42.9%)	737 (46.8%)	
	Unknown	517 (19.6%)	260 (24.5%)	257 (16.3%)	
ER status of primary tumor	Positive	1709 (64.8%)	591 (55.6%)	1118 (71.0%)	<0.001
	Negative	922 (35.0%)	468 (44.1%)	454 (28.8%)	
	Unknown	6 (0.2%)	3 (0.3%)	3 (0.2%)	
ER-positive staining percentage	0–9	1044 (39.6%)	504 (47.5%)	540 (34.3%)	<0.001
	10–54	264 (10.0%)	105 (9.9%)	159 (10.1%)	
	55–94	651 (24.7%)	205 (19.3%)	446 (28.3%)	
	95–100	519 (19.7%)	174 (16.4%)	345 (21.9%)	
	Unknown	159 (6.0%)	74 (7.0%)	85 (5.4%)	
PR status of primary tumor	Positive	1331 (50.5%)	452 (42.6%)	879 (55.8%)	<0.001
	Negative	1286 (48.8%)	602 (56.7%)	684 (43.4%)	
	Unknown	20 (0.8%)	8 (0.8%)	12 (0.8%)	
PR-positive staining percentage	0–9	1671 (63.4%)	713 (67.1%)	958 (60.8%)	0.003
	10–54	359 (13.6%)	118 (11.1%)	241 (15.3%)	
	55–94	330 (12.5%)	120 (11.3%)	210 (13.3%)	
	95–100	106 (4.0%)	42 (4.0%)	64 (4.1%)	
	Unknown	171 (6.5%)	69 (6.5%)	102 (6.5%)	
HR status of primary tumor	Positive	1757 (66.6%)	618 (58.2%)	1139 (72.3%)	<0.001
	Negative	868 (32.9%)	440 (41.4%)	428 (27.2%)	
	Unknown	12 (0.5%)	4 (0.4%)	8 (0.5%)	
Neoadjuvant chemotherapy	Yes	1175 (44.6%)	495 (46.6%)	680 (43.2%)	0.078
	No	1462 (55.4%)	567 (53.4%)	895 (56.8%)	
Neoadjuvant endocrine therapy	Yes	40 (1.5%)	12 (1.1%)	28 (1.8%)	0.179
	No	2597 (98.5%)	1050 (98.9%)	1547 (98.2%)	
Adjuvant chemotherapy	Yes	1301 (49.3%)	512 (48.2%)	789 (50.1%)	0.342
	No	1336 (50.7%)	550 (51.8%)	786 (49.9%)	
Adjuvant endocrine therapy	Yes	1376 (52.2%)	485 (45.7%)	891 (56.6%)	<0.001
	No	1261 (47.8%)	577 (54.3%)	684 (43.4%)	
Adjuvant radiation therapy	Yes	1694 (64.2%)	708 (66.7%)	986 (62.6%)	0.032
	No	943 (35.8%)	354 (33.3%)	589 (37.4%)	

ER, estrogen receptor; HR, hormone receptor; PR, progesterone receptor.

**Table 2:** Multivariate logistic regression for HER2 status (HER2-low vs. HER2-0) in patients with recurrent metastatic breast cancer.

Effect		Odds Ratio Point Estimate	95% Wald Confidence Limits		*p*-Value
Initial stage	II vs. I	1.22	0.97	1.54	0.088
	III vs. I	1.42	1.11	1.81	0.005
ER in primary	Positive vs. negative	1.96	1.66	2.31	<0.001
Adjuvant XRT	Yes vs. no	0.79	0.67	0.94	0.007

ER, estrogen receptor; XRT, radiation therapy.

**Table 3:** Demographic and clinicopathological characteristics by HER2 status in patients with de novo stage IV breast cancer.

		All De Novo Patients (*n* = 1197), *n* (%)	HER2 Status		
Covariate			HER2-0 (*n* = 485), *n* (%)	HER2-Low (*n* = 712), *n* (%)	Univariate *p*-Value
Age	≤40	191 (16.0%)	75 (39.3%)	116 (60.7%)	0.4723
	41–59	582 (48.6%)	243 (41.8%)	339 (58.2%)	
	60–69	286 (23.9%)	119 (41.6%)	167 (58.4%)	
	≥70	138 (11.5%)	48 (34.8%)	90 (65.2%)	
	Mean ± standard deviation	54.46 ± 12.98	53.91 ± 12.59	54.84 ± 13.24	0.2117
Race/ethnicity	Black	149 (12.4%)	58 (38.9%)	91 (61.1%)	0.0989
	Hispanic	107 (8.9%)	55 (51.4%)	52 (48.6%)	
	Others	69 (5.8%)	30 (43.5%)	39 (56.5%)	
	White	872 (72.8%)	342 (39.2%)	530 (60.8%)	
Body mass index	Unknown	82			
	Normal	315 (28.3%)	127 (40.3%)	188 (59.7%)	0.9772
	Obese	412 (37%)	166 (40.3%)	246 (59.7%)	
	Overweight	368 (33%)	147 (39.9%)	221 (60.1%)	
	Underweight	20 (1.8%)	9 (45%)	11 (55%)	
	Mean ± standard deviation	29.00 ± 6.67	29.14 ± 6.85	28.90 ± 6.55	0.7227
Menopausal status	Unknown	390			
	Other	18 (2.2%)	2 (11.1%)	16 (88.9%)	0.0281
	Post	497 (61.6%)	177 (35.6%)	320 (64.4%)	
	Pre	292 (36.2%)	117 (40.1%)	175 (59.9%)	
Histology	Invasive ductal	935 (78.1%)	378 (40.4%)	557 (59.6%)	0.1975
	Invasive lobular	150 (12.5%)	60 (40%)	90 (60%)	
	Metaplastic	14 (1.2%)	10 (71.4%)	4 (28.6%)	
	Invasive mixed ductal/lobular	54 (4.5%)	19 (35.2%)	35 (64.8%)	
	Other	44 (3.7%)	18 (40.9%)	26 (59.1%)	
Nuclear grade	Unknown	210			
	1	71 (7.2%)	39 (54.9%)	32 (45.1%)	0.0224
	2	422 (42.8%)	159 (37.7%)	263 (62.3%)	
	3	494 (50.1%)	195 (39.5%)	299 (60.5%)	
Lymphovascular invasion	Unknown	773			
	Negative	211 (49.8%)	82 (38.9%)	129 (61.1%)	0.4363
	Positive	213 (50.2%)	75 (35.2%)	138 (64.8%)	
ER status of primary tumor	Unknown	3			
	Negative	264 (22.1%)	125 (47.3%)	139 (52.7%)	0.0088
	Positive	930 (77.9%)	357 (38.4%)	573 (61.6%)	
ER-positive staining percentage	Unknown	45			
	0–9%	297 (25.8%)	138 (46.5%)	159 (53.5%)	0.0470
	10–54%	118 (10.2%)	52 (44.1%)	66 (55.9%)	
	55–94%	371 (32.2%)	141 (38%)	230 (62%)	
	95–100%	366 (31.8%)	135 (36.9%)	231 (63.1%)	
	Mean ± standard deviation, median, %	61.17 ± 40.70, 85	56.67 ± 41.65, 80	64.23 ± 39.78, 90	0.0015
PR status of primary tumor	Unknown	2			
	Negative	449 (37.6%)	192 (42.8%)	257 (57.2%)	0.2003
	Positive	746 (62.4%)	291 (39%)	455 (61%)	
PR-positive staining percentage	Unknown	56			
	0–9%	576 (50.5%)	243 (42.2%)	333 (57.8%)	0.2605
	10–54%	252 (22.1%)	94 (37.3%)	158 (62.7%)	
	55–94%	236 (20.7%)	88 (37.3%)	148 (62.7%)	
	95–100%	77 (6.7%)	36 (46.8%)	41 (53.2%)	
	Mean ± standard deviation, median, %	29.39 ± 35.95, 8	28.96 ± 36.28, 5	29.68 ± 35.75, 10	0.2623
HR status of primary tumor	Unknown	2			
	Negative	243 (20.3%)	117 (48.1%)	126 (51.9%)	0.0059
	Positive	952 (79.7%)	366 (38.4%)	586 (61.6%)	

ER, estrogen receptor; HR, hormone receptor; PR, progesterone receptor. Values are *n* (%) except where otherwise indicated.

**Table 4:** Multivariate logistic regression for HER2 status (HER2-low vs. HER2-0) in de novo stage IV group.

Effect		Odds Ratio Point Estimate	95% Wald Confidence Limits		*p*-Value
Nuclear grade	2 (vs. 1)	2.016	1.214	3.348	0.0068
	3 (vs. 1)	1.869	1.132	3.084	0.0145

**Table 5:** Multivariate Cox proportional hazards model for overall survival in the recurrent metastatic breast cancer group.

Parameter		*p*-Value	Hazard Ratio	95% CI	
Race	Black vs. White	0.0078	1.210	1.051	1.392
	Hispanic vs. White	0.6497	0.963	0.816	1.135
	Other vs. White	0.0242	0.775	0.621	0.967
ER staining	10–54% vs. 0–9%	<0.001	0.689	0.585	0.810
	55–94% vs. 0–9%	<0.0001	0.424	0.366	0.491
	≥95% vs. 0–9%	<0.0001	0.439	0.376	0.513
PR staining	10–54% vs. 0–9%	0.688	0.967	0.822	1.138
	55–94% vs. 0–9%	0.0006	0.727	0.607	0.872
	≥95% vs. 0–9%	0.0296	0.728	0.547	0.969
HER2-negative	1+/2+FISH− vs. 0	0.0194	0.890	0.807	0.981

**Table 6:** Multivariate Cox proportional hazards model for overall survival in de novo stage IV group.

Parameter		*p*-Value	Hazard Ratio	95% CI	
Race	Black vs. White	0.0006	1.483	1.183	1.859
	Hispanic vs. White	0.0342	0.740	0.559	0.978
	Others vs. White	0.0093	0.565	0.367	0.869
Histology	Invasive ductal vs. mixed ductal/lobular	0.3452	0.894	0.707	1.129
	Metaplastic vs. ductal/lobular	0.0010	3.146	1.592	6.218
	Other vs. mixed ductal/lobular	0.0007	2.357	1.434	3.874
Nuclear grade	2 vs. 1	0.3467	1.197	0.823	1.740
	3 vs. 1	0.0079	1.673	1.145	2.446
ER staining	10–54% vs. 0–9%	<0.0001	0.484	0.361	0.650
	55–94% vs. 0–9%	<0.0001	0.443	0.347	0.567
	≥95% vs. 0–9%	<0.0001	0.413	0.318	0.536
PR staining	10–54% vs. 0–9%	0.9482	0.993	0.795	1.239
	55–94% vs. 0–9%	0.0104	0.718	0.558	0.925
	≥95% vs. 0–9%	0.0048	0.527	0.338	0.822
HER2-negative	1+/2+FISH− vs. 0	0.0025	0.774	0.656	0.913

**Table 7:** HER2 status of primary and distant tumors in patients with recurrent and de novo stage IV metastatic breast cancer.

		HER2 Status of Metastasis		
		0	1+/2+FISH−	Total
Cases with recurrent metastatic breast cancer				
HER2-negative status of primary tumor at diagnosis	0	157	78	235 (38.6%)
	1+/2+FISH−	158	216	374 (61.4%)
	Total	315 (51.7%)	294 (48.3%)	609 (100%)
		McNemar’s test *p* < 0.0001		
Cases with de novo metastatic breast cancer				
HER2-negative status of primary tumor at diagnosis	0	159	16	175 (45.1%)
	1+/2+FISH−	35	178	213 (54.9%)
	Total	194 (50.0%)	194 (50.0%)	388 (100%)
		McNemar’s test *p* = 0.0078		

**Table 8:** Site of metastasis.

	Metastasis Site		*n*	%
Recurrent metastatic breast cancer (*n* = 609)	Bone		247	40.6%
	Central nervous system		20	3.3%
	Liver		118	19.4%
	Lymph node		76	12.5%
	Lung/pleura		107	17.6%
	Others		14	2.3%
	Skin/soft tissue		27	4.4%
	Tissue type	Visceral	229	37.6%
		Non-visceral	380	62.4%
De novo stage IV (*n* = 388)	Bone		215	55.4%
	Central nervous system		2	0.5%
	Liver		71	18.3%
	Lymph node		38	9.8%
	Lung/pleura		34	8.8%
	Others		20	5.2%
	Skin/soft tissue		8	2.1%
	Tissue type	Visceral	116	29.9%
		Non-visceral	272	70.1%

**Table 9:** HER2 status of the primary tumor at diagnosis according to the site of metastasis in patients with recurrent metastatic breast cancer.

			HER2 Status of Metastasis		
			0	1+/2+FISH−	Total
Cases with visceral metastasis					
HER2-negative status of primary tumor at diagnosis		0	61	25	86 37.5%
		1+/2+FISH−	60	83	143 62.5%
		Total	121 (52.8%)	108 (47.2%)	229 (100%)
			McNemar’s test *p* < 0.0001		
Cases with non-visceral metastasis					
HER2-negative status of primary tumor at diagnosis		0	96	53	149 39.2%
		1+/2+FISH−	98	133	231 60.8%
		Total	194 (51.1%)	186 (48.9%)	380 (100%)
			McNemar’s test *p* = 0.0003		

**Table 10:** HER2 status of the primary tumor and metastatic site in patients with de novo metastatic breast cancer by the site of metastasis.

			HER2 Status of Metastasis		
			0	1+/2+FISH−	Total
Cases with visceral metastasis					
HER2-negative status of primary tumor at diagnosis		0	47	4	51 44.0%
		1+/2+FISH−	9	56	65 56.0%
Total			56 (48.3%)	60 (51.7%)	116 (100%)
			McNemar’s test *p* = 0.1655		
Cases with non-visceral metastasis					
HER2-negative status of primary tumor at diagnosis		0	112	12	149 39.2%
		1+/2+FISH−	26	122	231 60.8%
Total			138 (50.7%)	134 (49.3%)	272 (100%)
			McNemar’s test *p* = 0.0231		

## Data Availability

The data that support the findings of this study are available on reasonable request to the corresponding author. The data is not publicly available to protect the privacy of study participants.

## Supplementary Materials

The following supporting information can be downloaded at: https://www.mdpi.com/article/10.3390/cancers18020253/s1, Table S1: Overall 2- and 5-year landmark survival rates by demographic and clinicopathological characteristics among the recurrent metastatic breast cancer group; Table S2: Overall 1-, 2-, 3-, and 5-year landmark survival rates by demographic and clinicopathological characteristics among the de novo metastatic breast cancer group.
