# Supplementary material for: Prevalence and Outcomes of HER2-Low Versus HER2-0 Status in Patients with Metastatic Breast Cancer
Source: Cancers (Basel). 2026 Jan 14;18(2):253. doi: 10.3390/cancers18020253 (PMC12838560; doi:10.3390/cancers18020253)
Supplement: Supplementary file 1 [file cancers-18-00253-s001.zip › cancers-4063843-supplementary.pdf]

Supplement Table S1. Overall 2 and 5-Year Landmark Survival Rates by Demographic and Clinicopathological Characteristics Among the Recurrent Metastatic Breast Cancer Group

Covariate		Year after diagnosis of recurrence	Overall survival rate (95% CI)	p value
Race/ethnicity	Black	2	0.301(0.248-0.356)	<.0001
		5	0.083(0.049-0.128)	
	Hispanic	2	0.461(0.394-0.526)	
		5	0.163(0.110-0.224)	
	Others	2	0.504(0.413-0.589)	
		5	0.206(0.117-0.313)	
	White	2	0.455(0.431-0.479)	
		5	0.139(0.120-0.159)	
ER status of primary tumor	Negative	2	0.222(0.194-0.252)	<.0001
		5	0.045(0.030-0.064)	
	Positive	2	0.559(0.533-0.584)	
		5	0.189(0.165-0.214)	
ER positive staining percentage	0%-9%	2	0.236(0.209-0.264)	<.0001
		5	0.042(0.028-0.060)	
	10%-54%	2	0.392(0.328-0.456)	
		5	0.098(0.058-0.150)	
	55%-94%	2	0.644(0.603-0.682)	
		5	0.233(0.192-0.276)	
	95%-100%	2	0.643(0.596-0.687)	
		5	0.236(0.189-0.286)	
PR status of primary tumor	Negative	2	0.275(0.249-0.302)	<.0001
		5	0.061(0.046-0.079)	
	Positive	2	0.603(0.574-0.631)	
		5	0.215(0.186-0.245)	
PR positive staining percentage	0%-9%	2	0.355(0.331-0.380)	<.0001
		5	0.089(0.073-0.107)	
	10%-54%	2	0.599(0.541-0.652)	
		5	0.179(0.129-0.235)	
	55%-94%	2	0.685(0.627-0.736)	
		5	0.308(0.242-0.377)	

Covariate		Year after diagnosis of recurrence	Overall survival rate (95% CI)	p value
	95%-100%	2	0.636(0.526-0.727)	
		5	0.354(0.239-0.471)	
HR status of primary tumor	Negative	2	0.218(0.189-0.248)	<.0001
		5	0.045(0.030-0.065)	
	Positive	2	0.552(0.526-0.576)	
		5	0.185(0.162-0.209)	
HER2 status	IHC 0	2	0.383(0.351-0.415)	<.0001
		5	0.106(0.082-0.133)	
	1+	2	0.480(0.449-0.511)	
		5	0.164(0.138-0.192)	
	2+/FISH-	2	0.469(0.418-0.519)	
		5	0.143(0.104-0.188)	
HER2 status	IHC 0	2	0.383(0.351-0.415)	<.0001
		5	0.106(0.082-0.133)	
	HER2-low	2	0.477(0.450-0.503)	
		5	0.158(0.136-0.182)	

Supplement Table S2. Overall 1, 2, 3 and 5-Year Landmark Survival Rates by Demographic and Clinicopathological Characteristics Among the *De Novo* Metastatic Breast Cancer Group

Covariate		Year after *de novo* diagnosis	Overall survival rate (95% CI)	p value
Race/ethnicity	Black	1	0.855(0.786-0.903)	<.0001
		2	0.494(0.408-0.573)	
		3	0.355(0.276-0.436)	
		5	0.175(0.112-0.248)	
	Hispanic	1	0.834(0.747-0.894)	
		2	0.664(0.562-0.747)	
		3	0.547(0.444-0.640)	
		5	0.361(0.264-0.459)	
	Others	1	0.948(0.846-0.983)	
		2	0.798(0.655-0.886)	
		3	0.649(0.488-0.770)	
		5	0.495(0.327-0.642)	
	White	1	0.872(0.847-0.893)	

Covariate		Year after *de novo* diagnosis	Overall survival rate (95% CI)	p value
		2	0.692(0.658-0.723)	
		3	0.556(0.519-0.591)	
		5	0.316(0.280-0.353)	
Histology	Invasive Ductal	1	0.873 (0.849-0.893)	<.0001
		2	0.668(0.635-0.699)	
		3	0.530(0.494-0.564)	
		5	0.302(0.268-0.337)	
	Invasive Lobular	1	0.925(0.865-0.959)	
		2	0.740(0.656-0.807)	
		3	0.620(0.529-0.699)	
		5	0.379(0.287-0.470)	
	Metaplastic	1	0.464(0.193-0.699)	
		2	0.077(0.005-0.293)	
		3	0.000	
		5	0.000	
	Invasive mixed ductal/lobular	1	0.959(0.847-0.990)	
		2	0.854(0.718-0.928)	
		3	0.679(0.524-0.793)	
		5	0.365(0.219-0.513)	
	Other	1	0.653(0.485-0.778)	
		2	0.392(0.241-0.540)	
		3	0.307(0.170-0.456)	
		5	0.246(0.121-0.393)	
Nuclear grade	1	1	0.919(0.816-0.966)	<.0001
		2	0.817(0.694-0.894)	
		3	0.743(0.609-0.836)	
		5	0.470(0.327-0.600)	
	2	1	0.914(0.882-0.938)	
		2	0.793(0.749-0.831)	
		3	0.685(0.634-0.730)	
		5	0.403(0.348-0.457)	
	3	1	0.828(0.790-0.859)	

Covariate		Year after *de novo* diagnosis	Overall survival rate (95% CI)	p value
		2	0.540(0.493-0.585)	
		3	0.383(0.337-0.428)	
		5	0.199(0.161-0.239)	
Lymphovascular invasion	Negative	1	0.937(0.895-0.963)	.0013
		2	0.771(0.707-0.822)	
		3	0.685(0.617-0.744)	
		5	0.424(0.351-0.495)	
	Positive	1	0.894(0.843-0.929)	
		2	0.682(0.613-0.742)	
		3	0.484(0.411-0.552)	
		5	0.284(0.219-0.352)	
ER status of primary tumor	Negative	1	0.676(0.613-0.730)	<.0001
		2	0.305(0.246-0.365)	
		3	0.181(0.133-0.236)	
		5	0.090(0.053-0.138)	
	Positive	1	0.929(0.910-0.945)	
		2	0.774(0.744-0.801)	
		3	0.634(0.599-0.667)	
		5	0.371(0.335-0.408)	
ER positive staining percentage	0%-9%	1	0.695(0.637-0.745)	<.0001
		2	0.307(0.252-0.364)	
		3	0.205(0.157-0.257)	
		5	0.094(0.059-0.138)	
	10%-54%	1	0.870(0.793-0.919)	
		2	0.680(0.585-0.758)	
		3	0.527(0.429-0.616)	
		5	0.293(0.206-0.385)	
	55%-94%	1	0.943(0.913-0.963)	
		2	0.793(0.746-0.833)	
		3	0.631(0.576-0.682)	
		5	0.350(0.293-0.408)	
	95%-100%	1	0.948(0.918-0.968)	
		2	0.844(0.798-0.880)	

Covariate		Year after *de novo* diagnosis	Overall survival rate (95% CI)	p value
		3	0.723(0.667-0.771)	
		5	0.470(0.406-0.531)	
PR status of primary tumor	Negative	1	0.753(0.709-0.792)	<.0001
		2	0.448(0.399-0.496)	
		3	0.289(0.245-0.336)	
		5	0.147(0.111-0.188)	
	Positive	1	0.945(0.925-0.959)	
		2	0.806(0.774-0.835)	
		3	0.685(0.647-0.720)	
		5	0.409(0.367-0.451)	
PR positive staining percentage	0%-9%	1	0.809(0.774-0.840)	<.0001
		2	0.530(0.486-0.572)	
		3	0.382(0.340-0.425)	
		5	0.200(0.163-0.238)	
	10%-54%	1	0.925(0.884-0.952)	
		2	0.780(0.720-0.829)	
		3	0.666(0.599-0.724)	
		5	0.316(0.249-0.385)	
	55%-94%	1	0.938(0.896-0.964)	
		2	0.827(0.767-0.872)	
		3	0.701(0.631-0.761)	
		5	0.493(0.414-0.567)	
	95%-100%	1	0.986(0.908-0.998)	
		2	0.931(0.825-0.974)	
		3	0.846(0.713-0.921)	
		5	0.691(0.529-0.807)	
HR status of primary tumor	Negative	1	0.663(0.598-0.720)	<.0001
		2	0.292(0.232-0.354)	
		3	0.173(0.124-0.229)	
		5	0.089(0.051-0.140)	
	Positive	1	0.926(0.907-0.942)	
		2	0.766(0.736-0.793)	
		3	0.626(0.591-0.658)	

Covariate		Year after *de novo* diagnosis	Overall survival rate (95% CI)	p value
		5	0.365(0.329-0.401)	
HER2 status	IHC 0	1	0.838(0.800-0.869)	.0463
		2	0.615(0.566-0.659)	
		3	0.471(0.421-0.519)	
		5	0.286(0.239-0.335)	
	HER2-low	1	0.892(0.866-0.913)	
		2	0.703(0.666-0.737)	
		3	0.574(0.534-0.612)	
		5	0.325(0.285-0.365)	
